# Synteny Enabled Upgrade of the Galapagos Giant Tortoise Genome Improves Inferences of Runs of Homozygosity

**DOI:** 10.1002/ece3.71358

**Published:** 2025-04-25

**Authors:** Evelyn L. Jensen, Chiara Marchisio, Alexander Ochoa, Rachel Gray, Vanessa Parra, Joshua M. Miller, F. Gözde Çilingir, Adalgisa Caccone

**Affiliations:** ^1^ School of Natural and Environmental Sciences, Newcastle University Newcastle Upon Tyne UK; ^2^ Faculty of Health and Life Sciences Universitat Pompeu Fabra Barcelona Spain; ^3^ Department of Ecology and Evolutionary Biology Yale University New Haven Connecticut USA; ^4^ Biology Department University of Kentucky Lexington Kentucky USA; ^5^ Department of Biological Sciences MacEwan University Edmonton Canada; ^6^ Department of Evolutionary Biology and Environmental Studies University of Zurich Zurich Switzerland; ^7^ Swiss Federal Institute for Research WSL Birmensdorf Switzerland

**Keywords:** chelonian, F_ROH_, genome syteny

## Abstract

The utility and importance of whole‐genome sequences are recognized across various fields, including evolution and conservation. However, for some taxa, like extinct species, using methods to generate contiguous genomes that rely on high‐quality DNA is impossible. In such cases, an alternative may be to employ synteny‐based methods using a genome from a closely related taxon to generate more complete genomes. Here we update the reference genome for the Pinta Island Galapagos giant tortoise ( *Chelonoidis abingdonii* ) without conducting additional sequencing through rescaffolding against the most closely related chromosome‐level genome assembly, the Aldabra giant tortoise ( *Aldabrachelys gigantea* ). This effort resulted in a much more contiguous genome, CheloAbing_2.0, with an N50 that is two orders of magnitude longer and large reductions in L50 and the number of gaps. We then examined the impact of the CheloAbing_2.0 genome on estimates of runs of homozygosity (ROH) using genome resequencing data from 37 individual Galapagos giant tortoises from the 13 extant lineages to test the mechanisms by which a fragmented assembly may over‐ or underestimate the number and extent of ROH. The use of CheloAbing_2.0 resulted in individual estimates of inbreeding, including ROH proportion (F_ROH_), number (N_ROH_), and cumulative length (S_ROH_), that were statistically different from those derived from the earlier genome assembly. This improved genome will serve as a resource for future efforts focusing on the ecology, evolution, and conservation of this species group. More broadly, our results highlight that synteny‐based scaffolding is promising for generating contiguous genomes without needing additional data types.

## Graphical abstract

Here we update the reference genome for the Pinta Island Galapagos giant tortoise ( *Chelonoidis abingdonii* ) through rescaffolding against the most closely related chromosome‐level genome assembly, the Aldabra giant tortoise ( *Aldabrachelys gigantea* ). This effort resulted in a much more contiguous genome, CheloAbing_2.0, with an N50 that is two orders of magnitude longer, which allowed us to test the mechanisms by which a fragmented assembly may over‐ or underestimate the number and extent of ROH. Analysis of resequencing data of 13 individuals resulted in individual estimates of inbreeding, including ROH proportion (FROH), number (NROH), and cumulative length (SROH), that were statistically different from those derived from the earlier genome assembly.

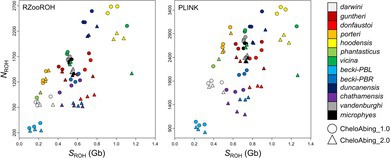

## 1 Introduction

The importance of chromosomal‐level reference genomes for conservation genetics is now well recognized (Brandies et al. 2019; Formenti et al. 2022; Paez et al. 2022). The ambition to produce reference genomes for all species has spawned a multitude of global efforts (e.g., Genome 10K Community of Scientists 2009; Bracken‐Grissom et al. 2014; Lewin et al. 2018), and steady progress is being made (e.g., Hotaling et al. 2021; Rhie et al. 2021). Unfortunately, some of the data that have been identified as key to producing the highest quality genomes (long reads, linked reads, optical maps, and proximity ligation; Rhie et al. 2021) require intact, high‐quality DNA or tissue, which will not be available for all organisms. In such cases (e.g., the extinct Tasmanian tiger, *Thylacinus cynocephalus* (Feigin et al. 2022)), researchers may have to make do with short reads and perhaps some long‐read data and employ bioinformatic approaches to achieve acceptable levels of contiguity. One commonly used approach is cross‐species scaffolding, whereby a reference‐assisted assembly is performed using de novo contigs from the focal species, and ordering and orienting them to a related high‐quality genome (Kim et al. 2013). Chelonians (turtles) in particular may be good candidates for cross‐species scaffolding (e.g., Mira‐Jover et al. 2024), as their genomes are known to be highly syntenic (Simison et al. 2020), meaning that the order of orthologous genes is conserved between different species (Shields 2001). For instance, Çilingir et al. (2022) reported finding 89%–94% of the Aldabra giant tortoise ( *Aldabrachelys gigantea* ) genome to be colinear with other chelonian species that diverged 50–70 million years ago (mya).

In the case of the Galapagos giant tortoises (*Chelonoidis* spp.), a radiation of closely related species, a reference genome was built for *Chelonoidis abingdonii* from Illumina short reads and PacBio SMRT long reads and published by Quesada et al. (2019). This resource constituted a major step forward in our understanding of genes key for age‐related disease and has enabled a suite of studies on these species, for example, evaluating the genetic impacts of conservation programs (Jensen et al. 2018), applying species delimitation models to clarify the taxonomic status of the 13 living taxa (Gaughran et al. 2024), and determining whether the species *C. phantasticus* , thought extinct for over a century, actually lives on (Jensen et al. 2022). However, the fragmented nature of the Galapagos giant tortoise reference genome (made up of > 10,000 scaffolds, Table 1) potentially limits its use for some key applications (Thomma et al. 2016), including evaluating recent genome‐wide inbreeding (Saremi et al. 2019) and other demographic histories (Ceballos et al. 2018) through runs of homozygosity (ROH).

**TABLE 1:** Contiguity and completeness measures for the original (CheloAbing_1.0) and upgraded (CheloAbing_2.0) Galapagos giant tortoise reference genome assemblies.

	CheloAbing_1.0		CheloAbing_2.0	
	Scaffold	Contig	Scaffold	Contig
# segments	10,618	65,418	3849	39,188
Largest segment	10,495,589	725,572	374,107,374	926,504
Total length	2,300,742,654	2,169,550,878	2,256,468,705	2,178,642,314
N50	1,277,207	73,186	148,185,065	119,261
N90	337,476	18,320	33,251,884	31,472
L50	529	8706	5	5399
L90	1833	30,937	17	18,768
GC (%)	43.71	43.71	43.77	43.77
# N's	131,201,757	10,476	77,830,784	4395
Busco	C: 95.7%, F: 1.2%, M: 3.1%		C: 96.8%, F: 0.5%, M: 2.7%	

*Note:* All lengths are given in base pairs.

Abbreviations: C, complete; F, fragmented; M, missing.

ROH are identified as stretches of genome that are homozygous and arise when two copies of an identical‐by‐descent haplotype are brought together in an individual through inbreeding. Fragmented assemblies may be particularly unsuitable for assessing ROH, because a single true ROH may be spread over > 1 contig in the reference genome assembly, resulting in either an overestimation of the true number of ROH or an underestimation because an ROH is split across multiple contigs such that one or both fall below the threshold to be recognized as an ROH. This latter scenario may also lead to a slight underestimation of the overall length of ROH and the proportion of the genome in ROH (F_ROH_). The relationship between the sum total length of ROH (S_ROH_) and the number of ROH segments (N_ROH_) can provide information about the demographic history of the population an individual is from, since long ROH can indicate recent inbreeding and shorter segments indicate more historical occurrences of inbreeding, where the ROH have been broken down over time due to recombination (Ceballos et al. 2018).

Upgrading the Galapagos giant tortoise reference genome to a level of contiguity approaching the chromosomal‐level is highly desirable but far from straightforward. In an ideal world, to improve this reference genome, new data would be collected to follow current best practices (Rhie et al. 2021). In this case, the species the reference was made from, *C. abingdonii* , went extinct in 2012, and no tissues suitable for conformation capture sequencing (e.g., Hi‐C, Belton et al. 2012) were preserved. Yet the conserved nature of chelonian genomes offers the possibility of improving the Galapagos giant tortoise reference genome through rescaffolding against the most closely related chromosome‐level genome assembly, the Aldabra giant tortoises (divergence 40 mya, Quesada et al. 2019), without the need for additional sequencing.

In this study, we improve the Galapagos giant tortoise reference genome by taking advantage of synteny among chelonian genomes by scaffolding contigs against the Aldabra giant tortoise genome to produce a new assembly. We then explore the impacts of an improved genome assembly on the analysis of ROH across the 13 living taxa of Galapagos giant tortoise to test the mechanisms by which a fragmented assembly may over‐ or underestimate the number and extent of ROH and affect inferences of demographic history.

## 2 Methods

### 2.1 Correcting and Rescaffolding CheloAbing_1.0

We downloaded the Galapagos giant tortoise (CheloAbing_1.0, GenBank accession GCA_003597395.1; Quesada et al. 2019) and Aldabra tortoise reference genomes (AldGig_1.0, GenBank accession GCA_026122505.1; Çilingir et al. 2022) in FASTA format and excluded the mitochondrial genome scaffolds (Contig5024, Contig5286, Contig5733, Contig6349, and Contig8551 for Galapagos; CM047529.1 for Aldabra). The PacBio long reads and Illumina short reads used to assemble CheloAbing_1.0 originally were downloaded from the SRA (see Table S1 for accession numbers and Appendix S1 for all scripts used in the genome upgrade). Illumina short reads were trimmed (using “–trimns and –trimqualities”) and merged using AdapterRemoval version 2 (Schubert et al. 2016).

To identify possible misassemblies and incorrect scaffolding in CheloAbing_1.0, we used RagTag *correct* version 2.1 (Alonge et al. 2022) to align it (the query genome) to the AdlGig_1.0 genome (the reference genome). RagTag generates whole‐genome alignments between the reference and the query. When the query discordantly maps to the reference, the query file is broken without replacing or removing any sequences (Alonge et al. 2022). To assist with this process, the Illumina trimmed and merged short reads from SRA SRR6950583 and SRR6950584 (median 17× coverage) were aligned to the query genome during RagTag *correct* to verify break points based on exceptionally high (> 205×) or low coverage (< 5×). We then used the RagTag *scaffold* module to order and orient the corrected query sequences to the Aldabra reference. The ordered and oriented contigs along each scaffold were joined with stretches of 100 “*N*” characters to represent gaps of unknown true length, instead of the distance between them along the Aldabra scaffold. Contigs in the CheloAbing_1.0 genome that did not align were appended to the end of the resulting assembly. After this step and each subsequent refinement, we evaluated the length and contiguity of the new assembly using Quast version 5.2 (Gurevich et al. 2013); results are presented in Table S2.

We then filled gaps in the newly scaffolded alignment using all the existing PacBio long reads (estimated at 0.5× coverage) using TGS‐GapCloser version 1.2.1 (Xu et al. 2020), with errors in the reads corrected using Racon version 1.4.3 (Vaser et al. 2017). The resulting assembly was further gap‐filled using Sealer version 2.3.7 (Paulino et al. 2015) in two iterations. First, using trimmed and merged Illumina data from all SRA accessions as single‐end reads (estimated 80× coverage), with a Bloom filter size of 20G and k‐mer values of 29, 69, 99, and 129. Second, the resulting assembly was used as input for a second iteration of Sealer using the same parameters, but with the non‐merged paired‐end reads from the same accessions.

To identify potential contaminant contigs, we used BWA version 0.7.17 *mem* (Li 2013) to align the merged single‐end reads from all SRA accessions to the new draft assembly. We filtered the resulting BAM file using BamTools version 2.5.2 (Barnett et al. 2011) to only retain primary alignments with a map quality > = 30 and marked duplicates using Picard version 2.25.6 (Broad Institute 2019). The read coverage profile of each contig was then checked using Qualimap version 2.3 (Okonechnikov et al. 2016). After examining the distribution of coverage across the assembly (mean 77×, standard deviation 290×), we manually curated the assembly by discarding contigs with < 25× or > 200× mean read depth. Additional contaminants were identified and removed using the NCBI Foreign Contamination Screen version 0.5.0 (NCBI 2023).

We assessed the completeness of the assembly before and after this manual curation using BUSCO (v5) analysis of orthologs (Manni et al. 2021), with default parameters and the sauropsid lineage dataset (version odb10). We identified repetitive elements in the original and new assemblies by generating a de novo repeat library using RepeatModeler version 2.0.4 (Flynn et al. 2020), which was then used to soft mask the assembly using RepeatMasker version 4.1.5 (Smit et al. 2020). Finally, we mapped the annotation of CheloAbing_1.0 to the upgraded reference genome using Liftoff version 1.6.3 (Shumate and Salzberg 2021) and invoked the polishing module to re‐align exons. This original annotation was produced by NCBI using the Eukaryotic Genome Annotation Pipeline, which took advantage of RNA‐seq data for several Galapagos giant tortoise individuals as well as RNA and protein sequence data from other organisms.

### 2.2 Resequencing Data Assembly

We then aligned previously published whole‐genome resequencing data (Jensen et al. 2021, Jensen et al. 2022; NCBI Bioproject PRJNA761229) of Galapagos giant tortoises (*n* = 37) to both the original *C. abingdonii* reference genome (CheloAbing_1.0) and the upgraded reference genome (hereafter “CheloAbing_2.0”). The radiation of Galapagos giant tortoises consists of 13 living lineages, presently described as 12 species, with one species ( *C. becki* ) consisting of two lineages, PBL and PBR. For each lineage, the genomes of three individuals were included in this study, except for *C. phantasticus* , for which there is only a single living member. To align these data, we used the Paleomix bam pipeline version 1.3.7 (Schubert et al. 2014). Briefly, Paleomix is a wrapper program that calls upon other tools to carry out read trimming (AdapterRemoval, Schubert et al. 2016), alignment (BWA *mem*, Li 2013), and filtering of PCR duplicates (Picard, Broad Institute 2019). Resulting BAM files were filtered for a minimum MQ of 30, a maximum insert size of 800 bp between read pairs, and to retain only primary alignments using BamTools (Barnett et al. 2011). Variants were then detected on scaffolds with a length > 100 kb using bcftools *mpileup/call* (Danecek et al. 2021). Genotype calls were filtered using VCFtools version 0.1.16 (Danecek et al. 2011) to retain only those based on a depth of 6 or greater, with a genotype quality score of at least 18. SNPs were filtered to retain sites that were in non‐repetitive regions of the genome, were biallelic, had a minor allele count of 2, had a maximum mean site depth within 1 standard deviation of the mean across loci, and had no missing data. This filtering resulted in a dataset of 1,616,547 loci for CheloAbing_1.0 and 1,784,426 loci for CheloAbing_2.0.

### 2.3 ROH

We used two different methods to evaluate the presence of ROH using the two genome assemblies: the rule‐based method in PLINK (Purcell et al. 2007) and the model‐based method in RZooROH (Bertrand et al. 2019). Both PLINK and RZooROH use the same input of filtered genotype calls in vcf format.

Using PLINK version 1.90 (Purcell et al. 2007), we estimated F_ROH_, N_ROH_, and S_ROH_ statistics across samples. We characterized ROHs as tracts consisting of 50 contiguous homozygous genotypes, which were identified after using sliding windows of 50 contiguous SNPs, requiring a minimum ROH length of 100 kb, and allowing 1 heterozygous genotype per sliding window to account for possible genotyping or sequencing error. F_ROH_ was calculated by dividing S_ROH_ by the sum of the assembly size of contigs > 100 kb, excluding contigs with fewer than 50 SNPs.

The package RZooROH version 0.3.1 (Bertrand et al. 2019) was run in R version 3.6.3 and implements a hidden Markov model that can be given a set number of “classes” (*k*) of ROH that represent different age‐related instances of historical inbreeding and then partitions segments of the genome into these classes. Thus, this analysis gives information on the total proportion of the genome in ROH, along with the proportion of the genome in different classes of ROH, representing how many generations ago inbreeding occurred. We ran the analysis using three, five, seven, or ten classes, with *R*_*k*_ equal to base five, and compared the Bayesian information criterion (BIC) scores across models; for both genome versions, seven had the lowest BIC. F_ROH_ was calculated by dividing S_ROH_ by the sum of the assembly size of contigs > 100 kb, excluding contigs with fewer than 50 SNPs.

For both PLINK and RZooROH methodologies, we used the Wilcoxon signed rank test in the R version 4.1.3 base package to detect statistical differences in F_ROH_, N_ROH_, and S_ROH_ across CheloAbing_2.0 vs. CheloAbing_1.0 pairs of samples. We further employed this test for long (> 1 Mb) and short (> 100 kb, < 1 Mb) F_ROH_ estimates.

## 3 Results

By rescaffolding against the Aldabra giant tortoise genome, the newly upgraded *C. abingdonii* CheloAbing_2.0 genome is a dramatic improvement from the original assembly, with an N50 that is two orders of magnitude longer (Table 1). The *correct* step in RagTag broke the original 10,618 scaffolds in CheloAbing_1.0 into 12,200 segments, of which 4709 (2,258,206,411 bp) were scaffolded onto the Aldabra reference. The unplaced segments (*n* = 7491) constituted just 1.8% of the total assembly length. Gap‐filling was conducted using the existing PacBio long reads and Illumina short reads that were collected when the original CheloAbing_1.0 genome was produced. Despite the extremely low coverage (0.5×) of the PacBio reads, 20,992 gaps were filled, totaling 29,369,291 bp. Subsequent gap‐filling after two iterations with short reads filled an additional 1208 gaps. After aligning short reads back to this refined assembly, we identified 394 scaffolds with mean coverage > 200× and 3279 with coverage < 25×. These likely contaminant contigs, along with 11 others identified by the NCBI FCS, represented < 1% of the total genome length, and their removal did not negatively impact the BUSCO score, which remained 96.8% complete (Table S2). The number of fragmented BUSCOs was reduced from 87 in CheloAbing_1.0 to 34 in version 2.0, with the total number of complete single copy BUSCOs increasing by 100. Repetitive elements made up 39.38% of CheloAbing_1.0 and 40.72% of the CheloAbing_2.0 genome (Table S3) and were subsequently soft masked. The genome annotation liftoff identified 24,458 of the predicted protein‐coding genes, though it was unable to place 203 genes.

### 3.1 ROH Differences Between Genome Versions

**FIGURE 1:**
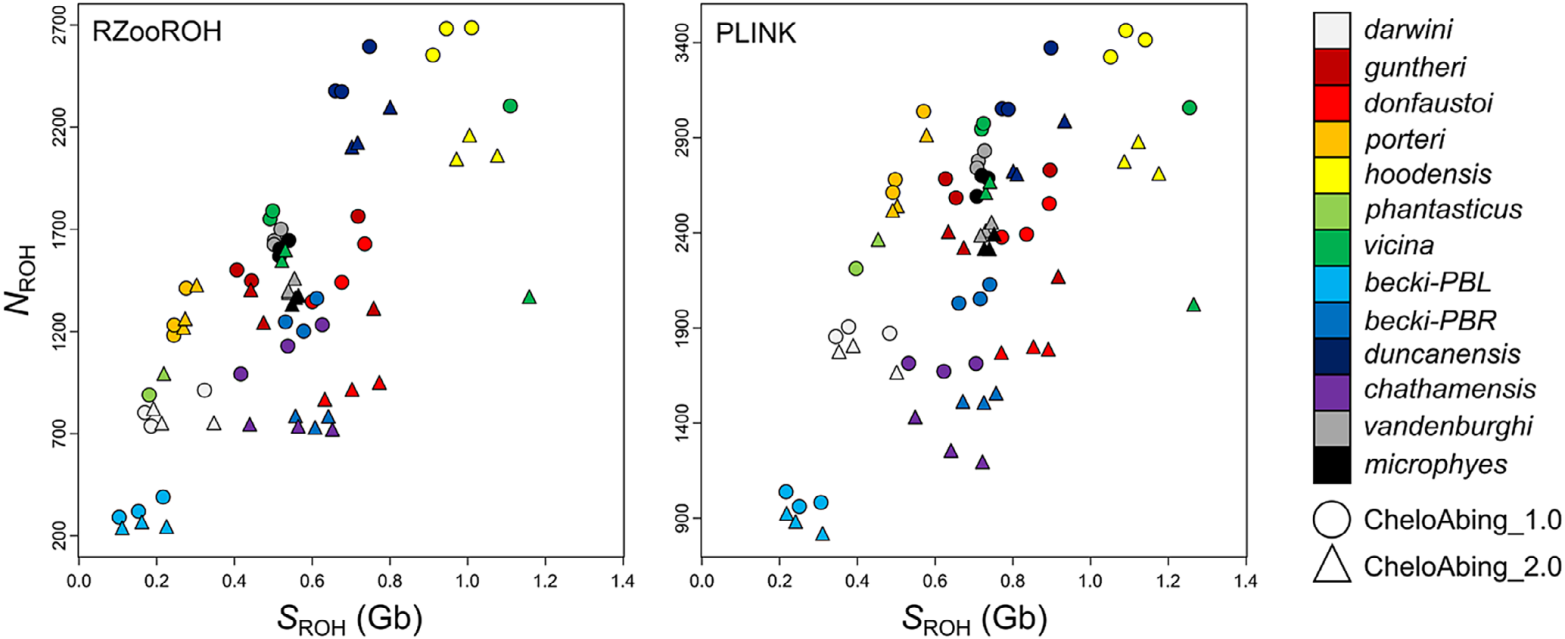
Comparisons of the number of ROH (N_ROH_) and sum total length of ROH (S_ROH_) for the original (CheloAbing_1.0, circles) and upgraded (CheloAbing_2.0, triangles) versions of the Galápagos giant tortoise reference genome for the RZooROH and PLINK analyses, for 1–3 individuals of each of the 13 living lineages (sample details provided in Table S4).

**FIGURE 2:**
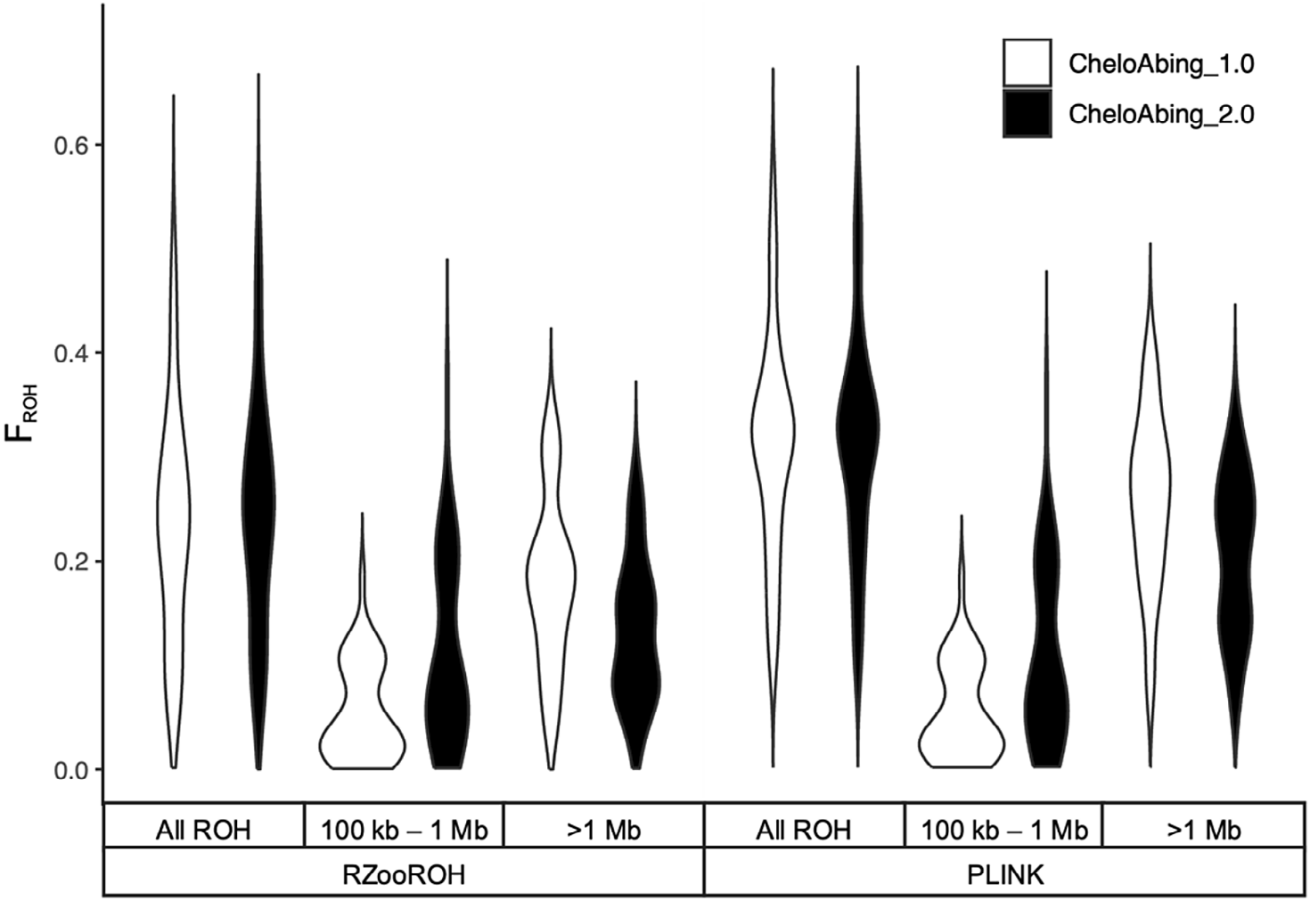
Violin plots of F_ROH_ showing the distribution of ROH segments by length class and overall for the original (CheloAbing_1.0) and upgraded (CheloAbing_2.0) versions of the Galápagos giant tortoise reference genome for the RZooROH and PLINK.

We evaluated the impact of the upgraded genome on ROH analyses using existing whole‐genome resequencing data from 37 individuals of Galapagos giant tortoises from 13 of the living lineages (Jensen et al. 2021, 2022). For both PLINK and RZooROH methodologies, we detected highly significant differences in F_ROH_, N_ROH_, and S_ROH_ between genome versions (*p* < 0.001 in all cases; Table 2 and Tables S4 and S5; Figure 1); specifically, CheloAbing_2.0 consistently produced smaller N_ROH_ and greater F_ROH_ and S_ROH_ estimates than Chelo_Abing_1.0 across pairs of samples. Moreover, CheloAbing_2.0 consistently produced greater long (> 1 Mb) and smaller short (> 100 kb, < 1 Mb) F_ROH_ estimates than Chelo_Abing_1.0 for the same sample set (*p* < 0.001 in all cases; Tables 2 and Tables S4 and S5; Figure 2). Although these differences in F_ROH_, N_ROH_, and S_ROH_ between genome versions are statistically significant, they are small in magnitude and lead to the same biological conclusions.

**TABLE 2:** Results from ROH analysis over 37 Galapagos giant tortoise individuals using the original (CheloAbing_1.0) and upgraded (CheloAbing_2.0) versions of the reference genome for RZooROH and PLINK, showing the average and range for each measure.

		CheloAbing_1.0	CheloAbing_2.0
RZooROH	F_ROH_ Total	0.234 (0.048–0.501)	0.246 (0.050–0.517)
	F_ROH_ Long	0.054 (0.001–0.184)	0.112 (0.003–0.370)
	F_ROH_ Short	0.179 (0.033–0.337)	0.134 (0.025–0.284)
	N_ROH_	1496 (294–2683)	1212 (243–2295)
	S_ROH_	517,341,638 (105,200,679—1,108,569,301)	550,187,058 (112,765,162—1,157,294,278)
PLINK	F_ROH_ Total	0.309 (0.098–0.566)	0.313 (0.098–0.565)
	F_ROH_ Long	0.053 (0.001–0.182)	0.108 (0.003–0.362)
	F_ROH_ Short	0.256 (0.084–0.399)	0.205 (0.066–0.347)
	N_ROH_	2453 (965–3460)	2090 (824–2986)
	S_ROH_	684,897,243 (217,934,000–1,253,170,000)	700,473,110 (219,303,000–1,263,723,426)

*Note:* F_ROH_, the average proportion of the genome in ROH; long ROH > 1 Mb; short ROH > 100 kb, < 1 Mb; N_ROH_ the average number of ROH; S_ROH_ the average total length of ROH in bp.

The interpretation of demographic history from the relationships between N_ROH_ and S_ROH_ is not strongly impacted by the genome version, despite greater values of N_ROH_ and smaller S_ROH_ for CheloAbing_1.0, as the interpretation is qualitative to begin with. For both PLINK and RZooROH analyses, there is a positive relationship between N_ROH_ and S_ROH_ (Figure 1), with some taxa, such as *becki*‐PBL, having low values for both measures, indicative of possible admixture in their recent history, and other taxa, such as *hoodensis* and *duncanensis*, having very high values, indicative of a history of bottlenecks, small population sizes, and inbreeding.

For 12 of the 13 lineages with multiple individuals represented in our dataset, there is little variation among individuals in either the length distribution of ROH (Figure S1), F_ROH_, N_ROH_, or S_ROH_ (Table S4). However, for *C. vicina* , one individual (LT_02) has substantially higher values than the other two from that taxon. This outlier behavior is not driven by a difference in coverage (all *C. vicina* have a mean depth of 12.1–12.6× for CheloAbing_2.0, Table S4) and may represent an unusually inbred member of the population.

## 4 Discussion

### 4.1 Success of the Synteny‐Enabled Rescaffolding

The improvement in the *C. abingdonii* reference genome enabled through rescaffolding to the Aldabra giant tortoise genome is remarkable, especially given that no new data were collected. By using only the short‐ and long‐read data from the original reference genome assembly, we have been able to increase the scaffold N50 > 100×, with the L90 (minimum number of scaffolds that produce 90% of the bases in the assembly) reduced from 1833 to just 17 (Table 1). These improvements in the scaffold‐level metrics are not matched at the contig level, with > 39,000 contigs remaining, and the N50 not even doubling in the new version, despite three rounds of gap‐filling after the rescaffolding. High repeat content is known to impact the level of assembly contiguity that can be achieved (Rhie et al. 2021). Thus, these persistent gaps may be due to the high proportion of the genome consisting of repetitive elements, which in turtles is typically around 42% (Simison et al. 2020), similar to the 41% found here.

### 4.2 Impact on ROH Inference

The main impact of the more contiguous CheloAbing_2.0 reference genome on ROH analyses was in the number of ROH segments detected. CheloAbing_1.0 had higher N_ROH_, suggesting that this version may be overestimating the number of ROH due to a single ROH being split across multiple contigs. Although the total F_ROH_ is statistically significantly higher in the upgraded genome version, the absolute difference is small, suggesting that this parameter is only slightly underestimated due to ROH being split across contigs and thus not meeting the thresholds to be called as an ROH.

Other studies have observed that F_ROH_ is robust to genome quality. In their study of ROH across 78 mammal species, Brüniche‐Olsen et al. (2018) did not find any correlation between genome N50 and F_ROH_, N_ROH_, or S_ROH_. However, this finding has not necessarily translated into confidence in applying ROH analyses when reference genome quality is low, as, intuitively, ROH could be under‐ or overestimated due to fragmentation. We hope that our direct comparison of ROH between genome versions will provide other researchers working with low contiguity genomes with the assurance that it is possible to estimate F_ROH_ and S_ROH_, but recognize that they may be systematically underestimated in a small but statistically significant way. Thus, statistical comparisons of F_ROH_, N_ROH_, and S_ROH_ between species should be made with caution, and the biological interpretations of any differences measured need to be considered. We note that parameter choice is particularly important when assessing ROH using a fragmented genome and that thresholds for minimum ROH length should be adjusted to reflect what is detectable. For example, here we have used 100 kb as a minimum threshold for ROH. If we had only considered ROH > 1 Mb, as is common when using SNP arrays (Meyermans et al. 2020), only 60% of the genome could have been assessed using this threshold for the CheloAbing_1.0 assembly, versus 96% at 100 kb minimum ROH length.

In general, the patterns of ROH across lineages are consistent with the findings of previous studies on the demographic history and genetic diversity of Galapagos giant tortoises. On Española Island, *C. hoodensis* has likely had a small population size for a long time (Jensen et al. 2021) and is fixed for a single mitochondrial haplotype (Caccone et al. 2002), so that this lineage has among the highest F_ROH_ is not surprising. Similarly, the Critically Endangered *C. donfaustoi* has declined in population size by 97% in the past three generations (Cayot et al. 2017), and the high incidence of both long and short ROH (CF samples in Figure S1) is consistent with this history. The comparatively low F_ROH_, N_ROH_, and S_ROH_ in *C. darwini* (also critically endangered, Cayot et al. 2022) is a positive sign for that species and is consistent with past findings of high genome‐wide heterozygosity and nucleotide diversity (Jensen et al. 2021).

### 4.3 Concluding Statements

Among the 357 species of turtles (Rhodin et al. 2021), only 38 species across 31 genera have a reference genome listed in NCBI as of March 2024. Our success with synteny‐based scaffolding suggests that reasonable‐quality genomes for the remaining species could be generated using standard sequencing data and a similar approach, without the need for costly additional data types. Cross‐species scaffolding does carry the risk of introducing misassemblies with impacts on downstream population genetic analyses (Prasad et al. 2022) and thus is not an appropriate approach for all taxa. However, in addition to turtles, some groups, such as birds (Zhang et al. 2014) and Dasyurid marsupials (Deakin 2018), are also known to have relatively conserved genomes, and synteny‐based approaches could perhaps be employed for them as well. ROH inference is not the only population genomic analysis that may be impacted by genome contiguity; structural variant analysis, linkage, copy number variation, and any method that uses a sliding window approach could also be biased. However, evaluating the impact of genome version on such methods was beyond the scope of the present study. The improved Galapagos giant tortoise reference genome is an important new asset that will enable a new era of analyses into their evolution, for example, potentially helping to resolve the biogeographic mystery of mito‐nuclear discordance (Jensen et al. 2022) and informing conservation through more detailed understanding of genome‐wide diversity patterns.

## Author Contributions

**Evelyn L. Jensen:** conceptualization (lead), data curation (lead), formal analysis (equal), funding acquisition (lead), investigation (equal), methodology (equal), project administration (lead), resources (equal), supervision (lead), visualization (lead), writing – original draft (lead), writing – review and editing (lead). **Chiara Marchisio:** conceptualization (supporting), formal analysis (supporting), investigation (supporting), writing – review and editing (supporting). **Alexander Ochoa:** conceptualization (supporting), formal analysis (equal), investigation (supporting), methodology (supporting), writing – original draft (supporting), writing – review and editing (equal). **Rachel Gray:** conceptualization (supporting), methodology (supporting), writing – review and editing (supporting). **Vanessa Parra:** methodology (supporting), writing – review and editing (supporting). **Joshua M. Miller:** conceptualization (supporting), methodology (supporting), writing – review and editing (supporting). **F. Gözde Çilingir:** conceptualization (supporting), methodology (supporting), writing – review and editing (supporting). **Adalgisa Caccone:** conceptualization (supporting), project administration (supporting), resources (equal), supervision (supporting), writing – review and editing (supporting).

## Conflicts of Interest

The authors declare no conflicts of interest.

## Supporting information


Appendix S1.

Appendix S2.

## Data Availability

This study made use of existing data available on the NCBI SRA under biosample SAMN07840320 and bioproject PRJNA761229. The CheloAbing_2.0 genome assembly has the NCBI accession number PKMU00000000. VCF files used for ROH analyses and the genome annotation file are available on Dryad DOI: 10.5061/dryad.sxksn03f6.
