# Supplementary material for: Synteny Enabled Upgrade of the Galapagos Giant Tortoise Genome Improves Inferences of Runs of Homozygosity
Source: Ecol Evol. 2025 Apr 25;15(4):e71358. doi: 10.1002/ece3.71358 (PMC12032190; doi:10.1002/ece3.71358)
Supplement: Supplementary file 1 — Appendix S1. [file ECE3-15-e71358-s002.docx]

Supplemental Materials

 Supplemental Figure 1. Stacked bar graphs of F_ROH_ showing the distribution of ROH segments by length class (short >100 kb and <1 Mb, long >1 Mb) for the original (CheloAbing_1.0) and upgraded (CheloAbing_2.0) versions of the Galapagos giant tortoise reference genome for the RZooROH and PLINK analyses for 37 individuals (each represented as pairs of bars along the x-axes) of Galapagos giant tortoise (sample details provided in Supplemental Table 4).

Supplemental table 1. List of SRA accession numbers for the PacBio and Illumina sequencing data used in for correcting, gap-filling and curating the reference genome.

| **PacBio** | **Illumina** |
| --- | --- |
| SRR6950571 | SRR6950581 |
| SRR6950572 | SRR6950582 |
| SRR6950573 | SRR6950583 |
| SRR6950574 | SRR6950584 |
| SRR6950575 | SRR6950585 |
| SRR6950576 | SRR6950586 |
| SRR6950577 | SRR6950587 |
| SRR6950578 | SRR6950588 |
| SRR6950579 | SRR6950589 |
| SRR6950580 | SRR6950590 |
| SRR6950591 | SRR6950598 |
| SRR6950592 | SRR6950599 |
| SRR6950593 | SRR6950600 |
| SRR6950594 | SRR6950615 |
| SRR6950595 | SRR6950616 |
| SRR6950596 | SRR6950617 |
| SRR6950601 | SRR6950618 |
| SRR6950602 | SRR6950619 |
| SRR6950603 | SRR6950620 |
| SRR6950604 | SRR6950621 |
| SRR6950605 | SRR6950622 |
| SRR6950606 | SRR6950623 |
| SRR6950607 | SRR6950624 |
| SRR6950608 |  |
| SRR6950609 |  |
| SRR6950610 |  |
| SRR6950611 |  |
| SRR6950612 |  |
| SRR6950613 |  |
| SRR6950614 |  |

Supplemental table 2. Contiguity and completeness measures for the genome after each step of synteny scaffolding, gap-filling and curation.

|  | Scaffold | Contig | Scaffold | Contig | Scaffold | Contig | Scaffold | Contig | Scaffold | Contig |
| --- | --- | --- | --- | --- | --- | --- | --- | --- | --- | --- |
|  | **CheloAbing1.0** | **CheloAbing1.0** | **After RagTag Scaffolding** | **After RagTag Scaffolding** | **After PacBio Gap Filling** | **After PacBio Gap Filling** | **After Illumina Gap Filling 2x** | **After Illumina Gap Filling 2x** | **After curation** | **After curation** |
| # contigs | 10618 | 65418 | 7549 | 66595 | 7549 | 44809 | 7526 | 43831 | 3849 | 39188 |
| # contigs (>= 0 bp) | 10618 | 65418 | 7549 | 66400 | 7549 | 44637 | 7529 | - | 3849 | - |
| # contigs (>= 1000 bp) | 10450 | 65148 | 7392 | 65981 | 7392 | 44262 | 7359 | 43273 | 3762 | 38939 |
| # contigs (>= 5000 bp) | 5637 | 46889 | 2703 | 47242 | 2713 | 32912 | 2631 | 32186 | 1163 | 31189 |
| # contigs (>= 10000 bp) | 4311 | 39422 | 1110 | 39459 | 1104 | 28522 | 1048 | 27924 | 492 | 27612 |
| # contigs (>= 25000 bp) | 3381 | 25959 | 273 | 25941 | 264 | 21231 | 255 | 20930 | 150 | 20898 |
| # contigs (>= 50000 bp) | 2966 | 14415 | 89 | 14389 | 86 | 14290 | 83 | 14195 | 72 | 14187 |
| Largest contig | 10495589 | 725572 | 378287534 | 725572 | 374207390 | 926504 | 374107374 | 926504 | 374107374 | 926504 |
| Total length | 2300742654 | 2169550878 | 2301207754 | 2169550479 | 2279450209 | 2196298653 | 2277650357 | 2195552274 | 2256468705 | 2178642314 |
| Total length (>= 0 bp) | 2300742654 | 2169550878 | 2301207754 | 2169550479 | 2279450209 | 2196298653 | 2277650529 | - | 2256468705 | - |
| Total length (>= 1000 bp) | 2300582124 | 2169302319 | 2301057713 | 2169262427 | 2279300168 | 2196044729 | 2277492275 | 2195297023 | 2256385145 | 2178489117 |
| Total length (>= 5000 bp) | 2291612800 | 2125401461 | 2292283994 | 2123922988 | 2270536262 | 2170251488 | 2268675083 | 2170295893 | 2251657682 | 2160182674 |
| Total length (>= 10000 bp) | 2281815604 | 2070086855 | 2280990459 | 2066612062 | 2259157264 | 2137912256 | 2257439732 | 2138877114 | 2246792122 | 2133728916 |
| Total length (>= 25000 bp) | 2267674380 | 1845531594 | 2268754032 | 1841394549 | 2246942966 | 2016116620 | 2245840990 | 2021923004 | 2241724910 | 2020654114 |
| Total length (>= 50000 bp) | 2252878727 | 1430476508 | 2262547547 | 1426009564 | 2241043914 | 1764266874 | 2240110863 | 1777372722 | 2239080834 | 1776879753 |
| N50 | 1277207 | 73186 | 149609123 | 72820 | 148256629 | 115217 | 148185065 | 118090 | 148185065 | 119261 |
| N90 | 337476 | 18320 | 28478836 | 18047 | 28100517 | 29076 | 28094189 | 29972 | 33251884 | 31472 |
| auN | 1700713 | 94431 | 178497767 | 94017 | 176657111 | 146637 | 176689513 | 150616 | 178347307 | 151708 |
| L50 | 529 | 8706 | 5 | 8742 | 5 | 5620 | 5 | 5470 | 5 | 5399 |
| L90 | 1833 | 30937 | 18 | 31147 | 18 | 19770 | 18 | 19254 | 17 | 18768 |
| GC (%) | 43.71 | 43.71 | 43.71 | 43.71 | 43.78 | 43.78 | 43.78 | 43.78 | 43.77 | 43.77 |
| # N's per 100 kbp | 5702.58 | 0.48 | 5721.64 | 0.49 | 3648.03 | 0.21 | 3604.68 | 0.21 | 3449.23 | 0.2 |
| # N's | 131201757 | 10476 | 131666857 | 10623 | 83155128 | 4612 | 82102077 | 4678 | 77830784 | 4395 |
| BUSCO Summary | C:95.7%[S:94.6%,D:1.1%],F:1.2%,M:3.1%,n:7480 | | |  |  |  | C:96.8%[S:95.9%,D:0.9%],F:0.5%,M:2.7%,n:7480 | | C:96.8%[S:95.9%,D:0.9%],F:0.5%,M:2.7%,n:7480 | |
| Complete BUSCOs (C) | 7158 |  |  |  |  |  | 7243 |  | 7241 |  |
| Complete and single-copy BUSCOs (S) | 7077 |  |  |  |  |  | 7176 |  | 7177 |  |
| Complete and duplicated BUSCOs (D) | 81 |  |  |  |  |  | 67 |  | 64 |  |
| Fragmented BUSCOs (F) | 87 |  |  |  |  |  | 34 |  | 34 |  |
| Missing BUSCOs (M) | 235 |  |  |  |  |  | 203 |  | 205 |  |
| Total BUSCO groups searched | 7480 |  |  |  |  |  | 7480 |  | 7480 |  |

Supplemental table 3. Details of repetitive elements identified by RepeatModeler and RepeatMasker.

|  |  | **CheloAbing_1.0** |  |  | **CheloAbing_2.0** |  |  |
| --- | --- | --- | --- | --- | --- | --- | --- |
|  |  | Number of elements | Length occupied | Percentage of sequence | Number of elements | Length occupied | Percentage of sequence |
| Retroelements |  | 1106668 | 358163070 bp | 15.57% | 1192314 | 367542224 bp | 16.29% |
| SINEs: |  | 301454 | 43590462 bp | 1.89% | 297650 | 34733769 bp | 1.54% |
| Penelope: |  | 0 | 0 bp | 0.00% | 0 | 0 bp | 0.00% |
| LINEs: |  | 676782 | 236081816 bp | 10.26% | 749459 | 247068732 bp | 10.95% |
|  | CRE/SLACS | 0 | 0 bp | 0.00% | 0 | 0 bp | 0.00% |
|  | L2/CR1/Rex | 519843 | 180718922 bp | 7.85% | 569211 | 189493372 bp | 8.40% |
|  | R1/LOA/Jockey | 1111 | 1142651 bp | 0.05% | 975 | 1006531 bp | 0.04% |
|  | R2/R4/NeSL | 9502 | 5731247 bp | 0.25% | 6549 | 6501359 bp | 0.29% |
|  | RTE/Bov-B | 20055 | 10321405 bp | 0.45% | 18183 | 9335164 bp | 0.41% |
|  | L1/CIN4 | 6492 | 2708899 bp | 0.12% | 5436 | 1978019 bp | 0.09% |
| LTR elements: |  | 128432 | 78490792 bp | 3.41% | 145205 | 85739723 bp | 3.80% |
|  | BEL/Pao | 0 | 0 bp | 0.00% | 0 | 0 bp | 0.00% |
|  | Ty1/Copia | 573 | 92051 bp | 0.00% | 147 | 20142 bp | 0.00% |
|  | Gypsy/DIRS1 | 109284 | 69438529 bp | 3.02% | 115841 | 73051303 bp | 3.24% |
|  | Retroviral | 16006 | 8059704 bp | 0.35% | 26098 | 11617275 bp | 0.51% |
|  |  |  |  |  |  |  |  |
| DNA transposons |  | 619862 | 141212793 bp | 6.14% | 609826 | 144083393 bp | 6.39% |
| hobo-Activator |  | 340210 | 67947333 bp | 2.95% | 352222 | 69309122 bp | 3.07% |
| Tc1-IS630-Pogo |  | 30405 | 4979966 bp | 0.22% | 31751 | 4907803 bp | 0.22% |
| En-Spm |  | 0 | 0 bp | 0.00% | 0 | 0 bp | 0.00% |
| MULE-MuDR |  | 0 | 0 bp | 0.00% | 0 | 0 bp | 0.00% |
| PiggyBac |  | 0 | 0 bp | 0.00% | 166 | 58024 bp | 0.00% |
| Tourist/Harbinger |  | 177394 | 56435267 bp | 2.45% | 161082 | 59143432 bp | 2.62% |
| Other (Mirage, P-element, Transib) | | 0 | 0 bp | 0.00% | 0 | 0 bp | 0.00% |
|  |  |  |  |  |  |  |  |
| Rolling-circles |  | 3812 | 525604 bp | 0.02% | 5681 | 678231 bp | 0.03% |
|  |  |  |  |  |  |  |  |
| Unclassified: |  | 2293209 | 394097849 bp | 17.13% | 2239878 | 395035494 bp | 17.51% |
|  |  |  |  |  |  |  |  |
| Total interspersed repeats: |  |  | 893473712 bp | 38.83% |  | 906661111 bp | 40.18% |
|  |  |  |  |  |  |  |  |
|  |  |  |  |  |  |  |  |
| Small RNA: |  | 51391 | 8104938 bp | 0.35% | 54680 | 7294850 bp | 0.32% |
|  |  |  |  |  |  |  |  |
| Satellites: |  | 942 | 484164 bp | 0.02% | 1657 | 709164 bp | 0.03% |
| Simple repeats: |  | 239149 | 9456701 bp | 0.41% | 228935 | 8745978 bp | 0.39% |
| Low complexity: |  | 35096 | 1659173 bp | 0.07% | 34572 | 1649827 bp | 0.07% |

Supplemental table 4. Sample information and results from ROH analysis for each of the 37 Galapagos giant tortoise individuals using the original (CheloAbing_1.0) and upgraded (CheloAbing_2.0) versions of the reference genome for RZooROH and PLINK.

|  |  |  |  |  | **Plink** | | | | | | | | | | **RZooROH** | | | | | | | | | |
| --- | --- | --- | --- | --- | --- | --- | --- | --- | --- | --- | --- | --- | --- | --- | --- | --- | --- | --- | --- | --- | --- | --- | --- | --- |
|  |  |  | **CheloAbing_1.0** | **CheloAbing_2.0** | **CheloAbing_1.0** | | | | | **CheloAbing_2.0** | | | | | **CheloAbing_1.0** | | | | | **CheloAbing_2.0** | | | | |
| **Individual** | **Island** | **Lineage** | **Mean Depth** | **Mean Depth** | **NROH** | **SROH** | **FROH** | **Long FROH** | **Short FROH** | **NROH** | **SROH** | **FROH** | **Long FROH** | **Short FROH** | **NROH** | **SROH** | **FROH** | **Long FROH** | **Short FROH** | **NROH** | **SROH** | **FROH** | **Long FROH** | **Short FROH** |
| AGO08 | Santiago | darwini | 14.3 | 12.1 | 1,907 | 378,268,000 | 0.171 | 0.007 | 0.164 | 1,809 | 390,031,631 | 0.174 | 0.020 | 0.154 | 739 | 187,280,828 | 0.085 | 0.008 | 0.077 | 753 | 214,419,244 | 0.096 | 0.022 | 0.074 |
| AGO20 | Santiago | darwini | 15.1 | 12.6 | 1,873 | 483,901,000 | 0.219 | 0.039 | 0.180 | 1,669 | 501,620,517 | 0.224 | 0.080 | 0.144 | 914 | 323,955,640 | 0.146 | 0.038 | 0.109 | 755 | 348,150,687 | 0.156 | 0.081 | 0.074 |
| AGO32 | Santiago | darwini | 18.5 | 15.5 | 1,855 | 345,606,000 | 0.156 | 0.001 | 0.155 | 1,778 | 353,736,743 | 0.158 | 0.005 | 0.153 | 805 | 170,724,168 | 0.077 | 0.001 | 0.076 | 824 | 193,149,809 | 0.086 | 0.006 | 0.080 |
| CAZ11 | Isabela | guntheri | 17.8 | 15.2 | 2,683 | 626,921,000 | 0.283 | 0.019 | 0.265 | 2,405 | 634,129,766 | 0.283 | 0.039 | 0.244 | 1,501 | 407,018,310 | 0.184 | 0.017 | 0.167 | 1,405 | 442,790,308 | 0.198 | 0.038 | 0.160 |
| CAZ15 | Isabela | guntheri | 16.8 | 14.3 | 2,583 | 653,899,000 | 0.295 | 0.036 | 0.259 | 2,322 | 673,708,919 | 0.301 | 0.071 | 0.230 | 1,448 | 444,742,800 | 0.201 | 0.037 | 0.163 | 1,245 | 475,353,715 | 0.212 | 0.070 | 0.143 |
| CAZ22 | Isabela | guntheri | 16.0 | 13.7 | 2,728 | 895,122,000 | 0.404 | 0.096 | 0.308 | 2,171 | 916,235,031 | 0.410 | 0.187 | 0.223 | 1,763 | 717,876,200 | 0.324 | 0.098 | 0.226 | 1,314 | 757,815,892 | 0.339 | 0.189 | 0.149 |
| CF03 | Santa Cruz | donfaustoi | 14.4 | 12.2 | 2,376 | 771,030,000 | 0.348 | 0.104 | 0.245 | 1,772 | 770,186,719 | 0.344 | 0.192 | 0.152 | 1,347 | 600,612,309 | 0.271 | 0.107 | 0.165 | 870 | 632,478,753 | 0.283 | 0.204 | 0.079 |
| CF06 | Santa Cruz | donfaustoi | 15.8 | 13.5 | 2,553 | 893,266,000 | 0.404 | 0.117 | 0.287 | 1,789 | 890,701,315 | 0.398 | 0.249 | 0.149 | 1,628 | 735,054,820 | 0.332 | 0.116 | 0.216 | 952 | 772,499,240 | 0.345 | 0.261 | 0.084 |
| CF09 | Santa Cruz | donfaustoi | 15.7 | 13.4 | 2,392 | 834,888,000 | 0.377 | 0.117 | 0.260 | 1,803 | 852,504,463 | 0.381 | 0.227 | 0.154 | 1,441 | 676,130,204 | 0.306 | 0.119 | 0.187 | 918 | 702,510,559 | 0.314 | 0.230 | 0.084 |
| CRU12 | Santa Cruz | porteri | 20.0 | 17.0 | 2,679 | 497,907,000 | 0.225 | 0.003 | 0.222 | 2,541 | 503,248,494 | 0.225 | 0.003 | 0.255 | 1,182 | 245,338,544 | 0.111 | 0.002 | 0.123 | 1,221 | 270,239,570 | 0.121 | 0.007 | 0.116 |
| CRU14 | Santa Cruz | porteri | 17.9 | 15.1 | 2,611 | 491,900,000 | 0.222 | 0.003 | 0.219 | 2,517 | 491,283,151 | 0.220 | 0.008 | 0.217 | 1,412 | 276,980,187 | 0.111 | 0.002 | 0.109 | 1,428 | 303,903,779 | 0.136 | 0.007 | 0.114 |
| CRU38 | Santa Cruz | porteri | 16.9 | 14.3 | 3,036 | 570,826,000 | 0.258 | 0.002 | 0.256 | 2,912 | 578,202,612 | 0.258 | 0.006 | 0.214 | 1,232 | 245,955,559 | 0.125 | 0.003 | 0.108 | 1,263 | 274,336,022 | 0.123 | 0.003 | 0.133 |
| ESP01 | Española | hoodensis | 18.5 | 15.6 | 3,460 | 1,089,780,000 | 0.492 | 0.105 | 0.370 | 2,774 | 1,085,537,536 | 0.485 | 0.191 | 0.294 | 2,550 | 909,988,762 | 0.427 | 0.111 | 0.316 | 2,159 | 1,003,979,661 | 0.449 | 0.209 | 0.240 |
| ESP02 | Española | hoodensis | 26.6 | 23.0 | 3,321 | 1,050,550,000 | 0.475 | 0.108 | 0.384 | 2,878 | 1,121,645,055 | 0.501 | 0.235 | 0.289 | 2,683 | 1,009,213,251 | 0.411 | 0.119 | 0.337 | 2,060 | 1,075,239,056 | 0.481 | 0.207 | 0.227 |
| ESP08 | Española | hoodensis | 16.7 | 13.9 | 3,411 | 1,139,520,000 | 0.515 | 0.115 | 0.399 | 2,711 | 1,174,142,916 | 0.525 | 0.197 | 0.304 | 2,679 | 944,587,617 | 0.456 | 0.109 | 0.302 | 2,043 | 970,629,955 | 0.434 | 0.247 | 0.233 |
| Fern07 | Fernandina | phantasticus | 38.3 | 34.5 | 2,212 | 397,285,000 | 0.180 | 0.001 | 0.178 | 2,365 | 454,098,861 | 0.203 | 0.005 | 0.198 | 891 | 182,056,563 | 0.082 | 0.002 | 0.081 | 995 | 219,467,807 | 0.098 | 0.006 | 0.092 |
| LP01 | Isabela | vicina | 14.2 | 12.1 | 2,943 | 719,564,000 | 0.325 | 0.016 | 0.309 | 2,610 | 730,128,962 | 0.326 | 0.049 | 0.278 | 1,750 | 492,067,806 | 0.222 | 0.017 | 0.206 | 1,547 | 522,477,310 | 0.234 | 0.049 | 0.185 |
| LP08 | Isabela | vicina | 14.8 | 12.6 | 2,973 | 724,458,000 | 0.327 | 0.016 | 0.312 | 2,668 | 740,798,863 | 0.331 | 0.049 | 0.282 | 1,789 | 499,134,412 | 0.226 | 0.015 | 0.211 | 1,599 | 531,542,163 | 0.238 | 0.047 | 0.191 |
| LT02 | Isabela | vicina | 14.9 | 12.5 | 3,055 | 1,253,170,000 | 0.566 | 0.182 | 0.384 | 2,025 | 1,263,723,426 | 0.565 | 0.362 | 0.203 | 2,301 | 1,108,569,301 | 0.501 | 0.184 | 0.317 | 1,371 | 1,157,294,278 | 0.517 | 0.370 | 0.147 |
| PBL_H126 | Isabela | becki-PBL | 13.5 | 11.4 | 1,042 | 217,934,000 | 0.098 | 0.015 | 0.084 | 929 | 219,303,000 | 0.098 | 0.025 | 0.073 | 294 | 105,200,679 | 0.048 | 0.014 | 0.033 | 243 | 112,765,162 | 0.050 | 0.026 | 0.025 |
| PBL_H130 | Isabela | becki-PBL | 14.2 | 11.9 | 965 | 252,129,000 | 0.114 | 0.029 | 0.085 | 886 | 242,412,948 | 0.108 | 0.035 | 0.073 | 323 | 154,616,401 | 0.070 | 0.030 | 0.040 | 271 | 163,139,554 | 0.073 | 0.042 | 0.031 |
| PBL_H61 | Isabela | becki-PBL | 13.4 | 11.0 | 986 | 307,420,000 | 0.139 | 0.045 | 0.094 | 824 | 311,531,482 | 0.139 | 0.073 | 0.066 | 393 | 217,826,695 | 0.098 | 0.047 | 0.052 | 249 | 226,671,004 | 0.101 | 0.074 | 0.027 |
| PBR_B125 | Isabela | becki-PBR | 15.3 | 13.1 | 2,031 | 661,232,000 | 0.299 | 0.073 | 0.226 | 1,516 | 671,446,033 | 0.300 | 0.169 | 0.131 | 1,248 | 531,784,209 | 0.240 | 0.075 | 0.165 | 789 | 557,246,132 | 0.249 | 0.174 | 0.075 |
| PBR_B152 | Isabela | becki-PBR | 16.5 | 14.0 | 2,053 | 715,980,000 | 0.324 | 0.097 | 0.227 | 1,510 | 725,458,996 | 0.324 | 0.195 | 0.129 | 1,202 | 578,482,299 | 0.261 | 0.102 | 0.160 | 733 | 607,657,020 | 0.272 | 0.200 | 0.072 |
| PBR_E61 | Isabela | becki-PBR | 14.9 | 12.5 | 2,129 | 740,575,000 | 0.335 | 0.092 | 0.243 | 1,559 | 756,712,826 | 0.338 | 0.200 | 0.138 | 1,362 | 612,016,146 | 0.277 | 0.093 | 0.183 | 787 | 641,861,838 | 0.287 | 0.208 | 0.078 |
| PZ03 | Pinzón | duncanensis | 21.4 | 18.0 | 3,369 | 897,415,000 | 0.406 | 0.026 | 0.380 | 2,986 | 932,333,299 | 0.417 | 0.069 | 0.347 | 2,591 | 747,390,870 | 0.338 | 0.023 | 0.314 | 2,295 | 800,219,589 | 0.358 | 0.074 | 0.284 |
| PZ09 | Pinzón | duncanensis | 21.4 | 18.3 | 3,050 | 772,255,000 | 0.349 | 0.017 | 0.332 | 2,723 | 800,722,176 | 0.358 | 0.049 | 0.309 | 2,375 | 660,019,738 | 0.298 | 0.018 | 0.280 | 2,102 | 701,985,529 | 0.314 | 0.055 | 0.259 |
| PZ70 | Pinzón | duncanensis | 23.9 | 20.5 | 3,047 | 788,072,000 | 0.356 | 0.019 | 0.337 | 2,707 | 810,045,009 | 0.362 | 0.056 | 0.306 | 2,372 | 675,951,946 | 0.305 | 0.021 | 0.284 | 2,123 | 716,851,187 | 0.320 | 0.060 | 0.260 |
| SCR05 | San Cristóbal | chathamensis | 17.4 | 14.6 | 1,716 | 532,409,000 | 0.241 | 0.062 | 0.179 | 1,434 | 549,274,210 | 0.246 | 0.113 | 0.132 | 993 | 417,140,542 | 0.188 | 0.062 | 0.126 | 748 | 439,891,395 | 0.197 | 0.116 | 0.081 |
| SCR10 | San Cristóbal | chathamensis | 20.3 | 17.5 | 1,673 | 622,637,000 | 0.281 | 0.094 | 0.187 | 1,259 | 640,984,466 | 0.286 | 0.164 | 0.122 | 1,130 | 537,846,034 | 0.243 | 0.096 | 0.147 | 738 | 564,384,368 | 0.252 | 0.171 | 0.081 |
| SCR22 | San Cristóbal | chathamensis | 18.0 | 15.5 | 1,714 | 705,297,000 | 0.319 | 0.118 | 0.201 | 1,199 | 721,798,460 | 0.323 | 0.211 | 0.112 | 1,233 | 626,036,523 | 0.283 | 0.119 | 0.164 | 723 | 652,085,130 | 0.291 | 0.217 | 0.074 |
| VA378 | Isabela | vandenburghi | 18.4 | 15.6 | 2,777 | 711,229,000 | 0.321 | 0.027 | 0.295 | 2,410 | 730,025,437 | 0.326 | 0.075 | 0.251 | 1,645 | 503,235,056 | 0.227 | 0.027 | 0.200 | 1,391 | 539,598,310 | 0.241 | 0.075 | 0.166 |
| VA423 | Isabela | vandenburghi | 18.4 | 15.5 | 2,829 | 727,261,000 | 0.329 | 0.031 | 0.298 | 2,455 | 745,038,670 | 0.333 | 0.077 | 0.256 | 1,700 | 520,123,190 | 0.235 | 0.030 | 0.205 | 1,460 | 554,750,846 | 0.248 | 0.079 | 0.169 |
| VA984 | Isabela | vandenburghi | 18.4 | 15.5 | 2,739 | 707,891,000 | 0.320 | 0.029 | 0.291 | 2,387 | 717,096,440 | 0.321 | 0.066 | 0.254 | 1,625 | 502,674,974 | 0.227 | 0.030 | 0.197 | 1,401 | 539,043,109 | 0.241 | 0.068 | 0.173 |
| VD02 | Isabela | microphyes | 17.9 | 15.1 | 2,591 | 707,534,000 | 0.320 | 0.034 | 0.285 | 2,317 | 726,415,309 | 0.325 | 0.078 | 0.247 | 1,569 | 516,618,863 | 0.233 | 0.035 | 0.198 | 1,333 | 548,444,136 | 0.245 | 0.083 | 0.162 |
| VD06 | Isabela | microphyes | 19.2 | 16.1 | 2,699 | 720,312,000 | 0.325 | 0.037 | 0.288 | 2,315 | 739,468,779 | 0.331 | 0.090 | 0.240 | 1,602 | 517,384,363 | 0.234 | 0.036 | 0.198 | 1,366 | 557,239,233 | 0.249 | 0.088 | 0.161 |
| VD08 | Isabela | microphyes | 17.2 | 14.6 | 2,686 | 735,755,000 | 0.332 | 0.044 | 0.288 | 2,393 | 751,772,544 | 0.336 | 0.086 | 0.251 | 1,646 | 540,004,802 | 0.244 | 0.046 | 0.198 | 1,377 | 564,809,784 | 0.252 | 0.087 | 0.166 |
| **Average** |  |  | **17.9** | **15.2** | **2,453** | **684,897,243** | **0.309** | **0.053** | **0.256** | **2,090** | **700,473,110** | **0.313** | **0.108** | **0.205** | **1,496** | **517,341,638** | **0.234** | **0.054** | **0.179** | **1,212** | **550,187,058** | **0.246** | **0.112** | **0.134** |

Supplemental table 5. Detailed results from the Wilcoxon signed rank tests comparing the F_ROH_, N_ROH_, and S_ROH_ values across CheloAbing_2.0 vs. CheloAbing_1.0 for the 37 pairs of samples.

| **CheloAbing_2.0 vs. CheloAbing_1.0 (*n* = 37)** | **Plink** |  | **RZooROH** |  |
| --- | --- | --- | --- | --- |
| **Metric** | ***V*-statistic** | ***P*-value** | ***V*-statistic** | ***P*-value** |
| NROH | 8 | 2.28E-07 | 24 | 8.08E-07 |
| SROH | 677 | 1.56E-08 | 703 | 1.46E-11 |
| FROH | 568 | 7.29E-04 | 667 | 7.24E-08 |
| Long FROH | 703 | 1.46E-11 | 703 | 1.46E-11 |
| Short FROH | 21 | 6.51E-09 | 29 | 2.53E-08 |
